# Virome of Three Termite Species from Southern Vietnam

**DOI:** 10.3390/v14050860

**Published:** 2022-04-21

**Authors:** Alexander G. Litov, Anna I. Zueva, Alexei V. Tiunov, Nguyen Van Thinh, Natalia V. Belyaeva, Galina G. Karganova

**Affiliations:** 1 Laboratory of Biology of Arboviruses, FSASI Chumakov Federal Scientific Center for Research and Development of Immune-and-Biological Products of RAS, 108819 Moscow, Russia; novosti-wxo@yandex.ru; 2 A.N. Severtsov Institute of Ecology and Evolution, 119071 Moscow, Russia; aizueva.ecologist@gmail.com (A.I.Z.); a_tiunov@mail.ru (A.V.T.); 3 Southern Branch, Russian–Vietnamese Tropical Scientific and Technological Center, Ho Chi Minh City 70001, Vietnam; thinh39b@gmail.com; 4 Division of Entomology, Faculty of Biology, Lomonosov Moscow State University, 119234 Moscow, Russia; natalia.belyaeva45@gmail.com; 5 Institute for Translational Medicine and Biotechnology, Sechenov University, 119991 Moscow, Russia; 6 Division of Virology, Faculty of Biology, Lomonosov Moscow State University, 119234 Moscow, Russia

**Keywords:** metagenomics, virome, *Chuviridae*, *Deltavirus*, *Dagazvirus*, Isoptera, tropical forest, social insects

## Abstract

Modern metagenomic approaches enable the effective discovery of novel viruses in previously unexplored organisms. Termites are significant ecosystem converters and influencers. As with the majority of tropical forest insects, termites are studied insufficiently, and termite virome remains especially understudied. Here, we studied the virome of lichenophagous and mycophagous termites (*Hospitalitermes bicolor*, *Macrotermes carbonarius* and *Odontotermes wallonensis*) collected in the Cat Tien National Park (Vietnam). We assembled four full genomes of novel viruses related to *Solemoviridae*, *Lispiviridae*, *Polycipiviridae* and *Kolmioviridae*. We also found several contigs with relation to *Chuviridae* and *Deltaflexiviridae* that did not correspond to complete virus genomes. All the novel viruses clustered phylogenetically with previously identified viruses of the termites. Deltaflexi-like contigs were identified in the fungi-cultivating *M. carbonarius* and showed homology with viruses recently discovered in the edible basidiomycete mushrooms.

## 1. Introduction

Modern metagenomic technologies have enabled the discovery of diverse RNA viruses in different species of vertebrates and invertebrates [1,2]. The search for novel viruses not only advances the knowledge of biodiversity but also enriches the understanding of the evolution of the viruses and mechanisms of their genome realization. For instance, the discovery of the termite HDV-like virus shifted our understanding of the evolution and origin of the deltaviruses [3], and several novel genome structures have been found for solemo-like viruses [1,2].

Termites (Blattodea, Isoptera) are keystone species in tropical ecosystems. Being the most important decomposers of dead organic matter, different species of termites exploit a wide range of feeding substrates. Higher termites from the family Termitidae are characterized by highest diet diversity, including wood, grass, lichens or fungi from fungal gardens cultivated in the nest [4].

As with the majority of tropical forest insects, termites are studied insufficiently [5], while termite virome remains a nearly unmapped area of insect ecology. Until now, wood-feeding termites have been the primary focus of viral and microbiological research [6], although termite feeding types should be considered to understand features of the termite virome [7].

For our investigation, we chose three species of higher termites: *Hospitalitermes bicolor* (Haviland), *Macrotermes carbonarius* (Hagen) and *Odontotermes wallonensis* (Wasmann). These species represent less virologically studied feeding types of termites, lichenophages and mycophages. *H. bicolor* (Nasutitermitinae) feed on epiphytes in the forest canopy and forage in large columns, formed by up to 500,000 individuals [8,9]. *M. carbonarius* and *O. wallonensis* (Macrotermitinae) are open-air foraging species that collect plant litter and wood debris to cultivate fungi in the fungal gardens [10]. Colonies of *M. carbonarius* can reach more than 60,000 individuals [11], while nests of *O. wallonensis* can harbor from 45,000 to 75,000 individuals [12].

Recently, the virome of 50 species of termites from Australia, Kenya, French Guiana, Cameroon and Indonesia was studied, and 67 novel viruses from different phylogenetic groups were identified [7]. In another recent work, the hepatitis delta-like virus was identified in the wood-feeding *Schedorhinotermes intermedius* [3].

In the current work, we assembled four full genomes of the novel viruses and obtained data indicating the presence of two more viruses in three hitherto unstudied species of termites from Southeast Asia.

## 2. Materials and Methods

### 2.1. Termites Collection

Termites were collected in the lowland monsoon tropical forest in the Cat Tien National Park (Vietnam) in December 2019. Worker and soldier specimens of *H. bicolor* and *O. wallonensis*, and soldier specimens of *M. carbonarius* (Table 1), were taken over several days near the “Lagerstroemia” plot (11.429° N, 107.4274° S) [11]. Termites were identified using morphological features [13]. The soil of the area can be classified as Skeletic Greyzemic Umbrisols (Clayic) developed from basalt and tuff [14]. The most abundant tree species in the canopy are *Lagerstroemia calyculata* and *Tetrameles nudiflora*, with an admixture of *Haldina cordifolia*, *Hopea odorata*, *Sindora siamensis* and *Afzelia xylocarpa*.

To avoid contamination with bacterial and fungal cells, insects were treated with nystatin saline solution (2,000,000 U of antimycotic per 1 l of saline) and further rinsed with distilled water. Termites were frozen at −20 °C to transport them to the laboratory (which took about 10 days). In the laboratory, insects were stored at −70 °C before molecular analysis.

### 2.2. High-Throughput Sequencing

Termite specimens were homogenized in the pools (Table 1) using Tissue Lyser 2 (Qiagen, Hilden, Germany) for 15 min with frequency 25 s^−1^. High-throughput sequencing and the data-processing pipeline were the same as described previously [15].

RNA was extracted using TRI Reagent LS (Sigma, St. Louis, MA, USA) according to the manufacturer’s manual. After the extraction, host rRNA was depleted using an NEBNext Globin and rRNA Depletion Kit (NEB, E7750S, Ipswich, MA, USA) according to the manufacturer’s instructions. The obtained RNA was used for library preparation without polyA-enrichment using a NEBNext Ultra II RNA Library Prep Kit for Illumina (NEB, E7770, Ipswich, MA, USA) according to the manufacturer’s instructions. Final libraries were sequenced (single-end, 250-nt reads) on a HiSeq1500 (Illumina, San Diego, CA, USA), and raw reads were deposited under the BioProject accession number PRJNA817887.

### 2.3. Virus Detection and Sanger Sequencing

RNA was extracted using TRI Reagent LS (Sigma-Aldrich, St. Louis, MA, USA), according to the manufacturer’s manual. Reverse transcription with random hexamer primers (R6) and MMLV RT kit (Evrogen, Moscow, Russia) was performed on an isolated matrix following the manufacturer’s instructions [15]. PCR was performed using virus-specific oligonucleotides with Evrogen Taq-polymerase (Evrogen, Moscow, Russia) following the manufacturer’s instructions.

### 2.4. Assembly, Analysis and Visualization

Adaptor sequences, bases with quality lower than Q30 and reads with length less than 35 nt were discarded using Trimmomatic v0.39 [16]. Filtered reads were used for *de novo* contig assembly by the SPAdes v3.13.0 program with spades pipeline and default parameters [17]. 

The resultant contigs were screened for virus-related sequences using the blastn program from BLAST v2.9.0+ package (with default settings) and nt database. Contigs containing virus-related hits were translated using SnapGene Viewer v.5.0.4 (with default settings), and open reading frames (ORF) were visualized and extracted manually. Extracted ORFs were further analyzed with blastp (using default settings) to identify viral proteins. Some contigs with very high identities to known human pathogens were filtered out as possible contaminations. When SPAdes failed to produce contigs resembling full-length genomes, the SeqMan v.7.0.0 program was used to assemble genomes with contigs as an input. Obtained virus sequences and sequence of the deltaflexi-like contig 17_N1 + N237 were deposited in the GenBank database (accession numbers ON082761-ON082766).

The protein sequences of the RNA-dependent RNA polymerase, polyprotein or HDV-ag were extracted from the contigs. These sequences, along with homologs, were aligned using MAFFT v7.310 [18]. The alignments were further processed using TrimAL v1.4. rev 15 [19] to remove ambiguously aligned regions, and then, maximum-likelihood phylogenetic trees were constructed using phyML 3.3.20200621 [20]. 

Trees were visualized with FigTree v.1.4.4. Genome structure of the obtained viruses was visualized using a custom Python script. All post processing of the images was performed with the GIMP v.2.10.24 program.

## 3. Results

### 3.1. High-Throughput Sequencing

Three pools of three species of termites were studied in the current work: one pool of *Macrotermes carbonarius*, one pool of *Hospitalitermes bicolor,* and one of *Odontotermes wallonensis* (Table 1). High-throughput sequencing resulted in 9.4–9.9 million reads (after filtering). No known termite viruses were identified in the pools. However, virus-like sequences were found in all of the studied termite species. We found several contigs with homology to the viral RNA-dependent RNA polymerases (RdRp), close to the members of the *Deltaflexiviridae*, *Solemoviridae*, *Chuviridae*, *Lispiviridae*, *Polycipiviridae* and *Kolmioviridae*.

In the study, a contig or group of contigs that met three followed criteria were considered a complete viral genome. Firstly, contigs must have ORFs with homology to the proteins of known viruses. Second, the length of the contigs must be similar to the length of the close relatives, according to the phylogenetic relationships. Third, total ORF count and their alignment in the contig must follow the same pattern as in the known viruses. 

### 3.2. Solemoviridae—Related Contigs

Classical members of the *Solemoviridae* family have a monopartite positive-sense RNA genome that is 4–6 kb in length, with 4–10 ORFs. Plants are the usual hosts for these viruses [21]. However, recent studies on the metagenomes of the various insects have discovered a novel group of solemoviridae-related viruses. Some of these novel viruses have shown serious differences in the genome structure compared to the classical members of the *Solemoviridae* family, for example, by having a different ORF count and/or by splitting the genome into two segments [2].

In the current work, we discovered two contigs related to abovementioned novel solemoviruses in the pool of the *Macrotermes carbonarius*. One of the contigs had homology to the solemoviridae-like RdRps, with ~53% amino acid identity to the closest relative (Mafsystermes virus). The second contig showed homology to the coat protein of the novel solemovirus, with ~47% amino acid identity to the Wifsystermes virus. Based on these data, we concluded that both contigs belonged to the single novel solemoviridae-like virus named Cat Tien Macrotermes solemo-like virus (CMSV).

**Figure 1:**
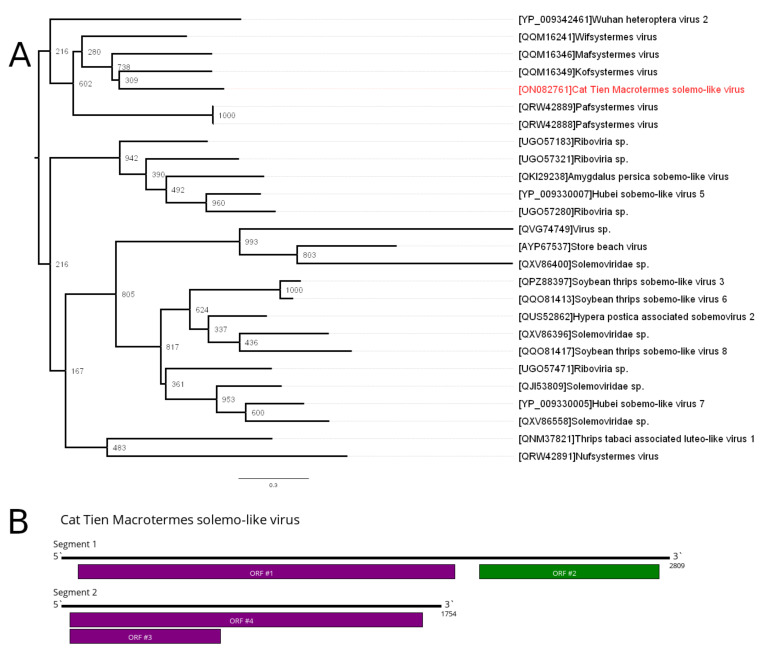
Phylogenetic relationships and genetic structure of the Cat Tien Macrotermes solemo-like virus (CMSV). (**A**) Phylogenetic relationships of the CMSV. Analysis was performed using amino acid sequences of viral polymerases, with 1000 bootstrap replicates. Tree is midpoint-rooted for the clarity only. Bootstrap support values are shown for each node. Scale bar represents the number of amino acid substitutions per site. CMSV virus is marked red. (**B**) Scheme of the CMSV virus genome, with RNA-dependent RNA polymerase is marked in green.

The genome of the CMSV has two segments, 2809 and 1754 nt in length, respectively (Figure 1B). Both segments encode two ORFs. The large segment encodes viral polymerase, while the ORFs on the second segment likely participate in viral coat protein production. Two ORFs on the second segment are located in the same frame and are divided by a single stop codon (UGA). As a similar genome pattern is frequently found in other solemo-like viruses, it is likely that the second ORF on the second segment is expressed by stop codon read-through [1,2,15].

According to the phylogenetic analysis based on the amino acids sequences of the RdRp (Figure 1A), CMSV groups together (although with low support) with the other solemo-like viruses previously identified in the termites, namely the Kofsystermes virus, Mafsystermes virus, Wifsystermes virus and Pafsystermes virus (detected in *Epikalotermes kempae*, *Epikalotermes kempae*, *Occasitermes* sp. 2 and *Schedorhinotermes intermedius*, respectively) [7]. 

### 3.3. Lispiviridae—Related Contig

According to the tenth release of the ICTV Virus Taxonomy, the family *Lispiviridae* (*Mononegavirales*) includes one genus composed of six viruses [22]. Their genome is a monopartitie, negative-sense RNA that is 9.9–14.5 kb in length, encoding 3–6 ORFs. All viruses infect various invertebrates. Currently, a number of related to the family *Lispiviridae* (but yet unclassified) viruses are known.

**Figure 2:**
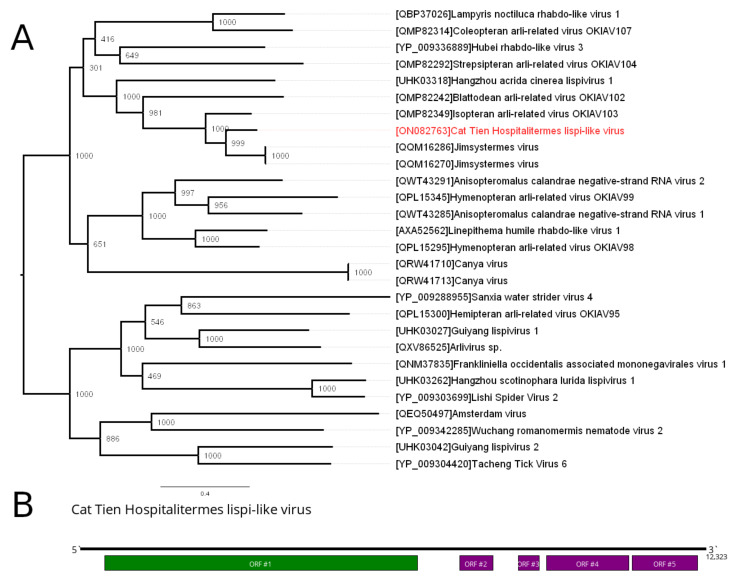
Phylogenetic relationships and genetic structure of the Cat Tien Hospitalitermes lispi-like virus (CHLV). (**A**) Phylogenetic relationships of the CHLV. Analysis was performed using amino acid sequences of viral polymerases, with 1000 bootstrap replicates. Tree is midpoint-rooted for the clarity only. Bootstrap support values are shown for each node. Scale bar represents the number of amino acid substitutions per site. CHLV is marked red. (**B**) Scheme of the CHLV genome, with RNA-dependent RNA polymerase is marked in green.

In the current work, we discovered a lispi-like contig in the pool of *Hospitalitermes bicolor*. The contig was 12323 nt long and contained five ORFs (Figure 2B). The largest ORF had homology to the RdRp of other lispi-like viruses, with the closest being the Jimsystermes virus (~66% amino acid identity). Based on these data, we concluded that we had a novel lispi-like virus and named it Cat Tien Hospitalitermes lispi-like virus (CHLV). Phylogenetic analysis based on the amino acid sequences of the RdRp showed that CHLV forms a close and well-supported group of two lispi-like viruses of the termites (Figure 2A): Jimsystermes virus [7], detected in *Occasitermes* sp., and Isopteran arli-related virus OKIAV103, detected in *Coptotermes* sp. [23].

### 3.4. Polycipiviridae—Related Contig

Viruses of the family *Polycipiviridae* have a monopartitie, positive-sense RNA genome that is 10–12 kb in length. It encodes one large polyprotein on the 3′ end of the genome. It contains RdRp, and the end products of this polyprotein play a major role in replication. Four or more ORFs on the 5′ end of the genome encode structural proteins and proteins with other functions. All members of the family were detected in the arthropods [24].

**Figure 3:**
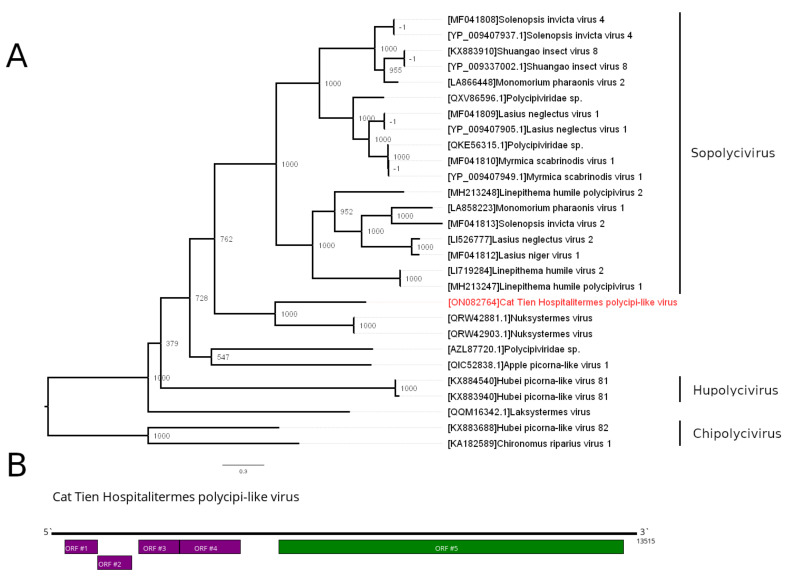
Phylogenetic relationships and genetic structure of the Cat Tien Hospitalitermes polycipi-like virus (CHPLV). (**A**) Phylogenetic relationships of the CHPLV. Analysis was performed using amino acid sequences of viral RdRp-encoding polyprotein, with 1000 bootstrap replicates. Bootstrap support values are shown for each node. Scale bar represents the number of amino acid substitutions per site. CHPLV is marked red. Genera of the *Polycipividae* family are marked on the right. (**B**) Scheme of the CHPLV genome, with RdRp-encoding polyprotein marked in green.

In the pool of *H. bicolor*, we identified a contig with usual ORF arrangement for polycipiviruses (Figure 3B): four smaller ORFs on the 5′ end and a large ORF on the 3′ end, which had significant similarities to the polycipiviruses RdRp-containing polyprotein (~43% amino acid identity to the Nuksystermes virus). Based on these data, we concluded that this contig belongs to the novel polycipi-like virus and named it Cat Tien Hospitalitermes polycipi-like virus (CHPLV). Phylogenetic analysis based on the amino acid sequences of the RdRp-containing polyprotein (Figure 3A) showed that CHPLV forms a group with the Nuksystermes virus, which was identified in *Heterotermes ferox* [7]. Out of all established genera, this group is closest to the genus *Sopolycivirus*, which infects ants.

### 3.5. Kolmioviridae—Related Contig

Hepatitis delta virus (HDV), of the genus *Deltavirus*, is a unique human pathogen. It has a small circular negative-stranded RNA genome of approximately 1.7 kb length that encodes one ORF: hepatitis delta antigen. Hepatitis delta antigen is expressed in two forms: large and small, both with unique functions. RNA of the HDV forms the ribozyme, allowing the self-cleavage and possibly self-ligation needed for virus replication. For the assembly and release of the infectious particles, HDV relies on the envelope proteins of the human hepatitis B virus (HBV) [25]. Whereas HDV is a human pathogen, several related viruses have recently been identified in fish, newts, birds, toads and even termites. All of these viruses contain ORF encoding the hepatitis delta antigen-like protein, and some of them have additional ORFs annotated, although without any functional proof of their expression [3].

In the current work, we discovered a single contig, 1810 nt in length, with significant similarities (72% identity with 25% query cover) to the previously discovered Termite HDV-like virus (THDV). The contig encoded several ORFs, and one of them showed similarities with hepatitis delta antigen sequences, with 52% identity to the closest relative THDV. Based on these data, we concluded that this contig belonged to the novel HDV-like virus and named it Cat Tien Odontotermes delta-like virus (CODLV). This contig had 123 nt direct repeats on both ends. Since it is known that deltaviruses have a circular genome, we believe that such repeat structure is an artifact of the sequencing and assembling of the circular genome. Thus, we deleted one of the repeating sequences to form a CODLV genome.

**Figure 4:**
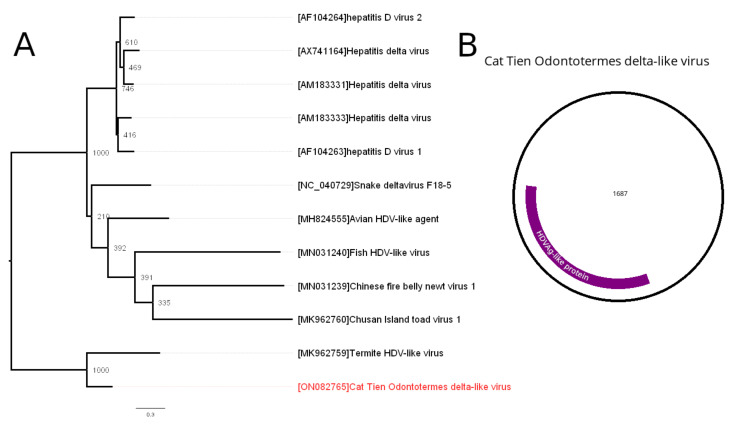
Phylogenetic relationships and genetic structure of the Cat Tien Odontotermes delta-like virus (CODLV). (**A**) Phylogenetic relationships of the CODLV. Analysis was performed using amino acid sequences of viral HDVAg, with 1000 bootstrap replicates. Bootstrap support values are shown for each node. Scale bar represents the number of amino acid substitutions per site. CODLV is marked red. (**B**) Scheme of the CODLV genome.

Phylogenetic analysis (Figure 4A), based on the amino acid sequence of the HDV antigen-like protein, clustered CODLV with the THDV—a single member of the *Dagazvirus* genus—detected in the *Schedorhinotermes intermedius* [3].

The genome of the CODLV was 1687 nt in length (Figure 4B). It showed an ORF encoding an HDV antigen-like protein, and several other ORFs could be identified in its genome. However, because they shared no homology with the additional ORFs found in the THDV, we decided not to annotate them as potential proteins, since more research is needed to prove that these novel ORFs are indeed expressed.

### 3.6. Other Virus-like Contigs

During the work, we also found several contigs with homologies to the viral RdRps (Table 2) that did not correspond to a complete genome of the closely related viruses. Moreover, all attempts to complete this contigs by amplification of the missing genome fragments using contig-specific oligonucleotides, followed by Sanger sequencing, failed. Thus, it is unclear if these contigs represent low-abundance viruses or endogenous viral elements in a host genome.

In the pool of the *Macrotermes carbonarius*, we found two contigs that were 4685 and 2470 nt in length, with homology to the Agaricus bisporus virus 9 RdRp-containing polyprotein. Agaricus bisporus virus 9 seemed to be close to the genus *Deltaflexivirus*. Generally, *Deltaflexivirus* members have a genome of about 8200 nt, with one large ORF encoding for polyprotein in the 5′end of the genome and 3–4 smaller ORFs close to the 3′end of the genome.

**Figure 5:**
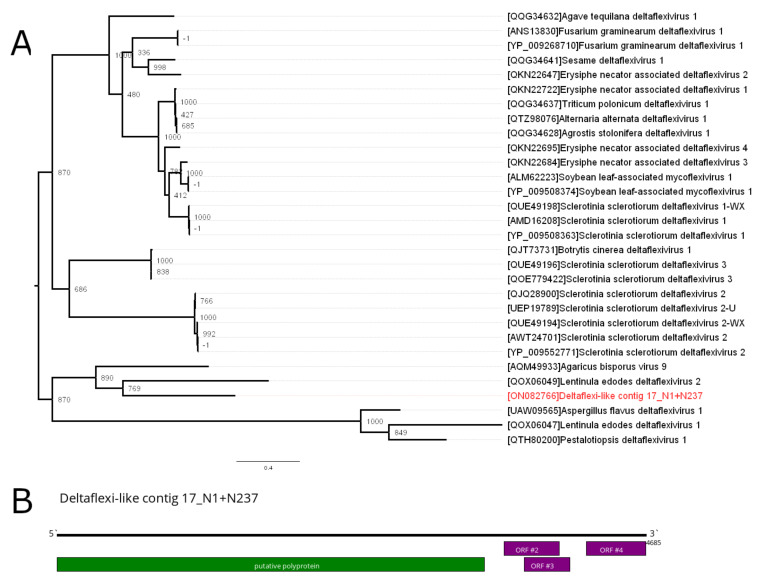
Phylogenetic relationships and genetic structure of the deltaflexi-like contig 17_N1 + N237. (**A**) Phylogenetic relationships of the sequences of contig-encoded polyprotein using amino acid sequence with 1000 bootstrap replicates. Bootstrap support values are shown for each node. Scale bar represents the number of amino acid substitutions per site. Deltaflexi-like contig 17N1 + N237 is marked red. (**B**) Scheme of the deltaflexi-like contig 17_N1 + N237.

Whereas contig 17_N1 + N237 had a similar structure to the *Deltaflexivirus* members (Figure 5B), its length was far below the usual length for *Deltaflexivirus* members. At the same time, 17_N195 + N540 contig had a homology 5′end of the *Deltaflexivirus* RdRp-containing polyprotein. Based on the assumption that both contigs belonged to the single virus, we attempted to PCR amplify the missing part of the genome. However, this attempt was unsuccessful.

We also found five contigs with homology to the polymerase of the various members of *Mononegavirales* in the *O. wallonensis* pool. These data may indicate the presence of at least one novel virus in the pool of *O. wallonensis.* However, no ORFs with homologies to non-RdRp proteins were found. Therefore, we are not ready to draw any conclusions on the viral nature of these contigs.

## 4. Discussion

In addition to the great ecological and economic significance of termites, they are attracting attention in biomedical research due to their eusociality. Infection transmission through the social contact network has become one of the most important health issues in the modern world [26], and there are clear similarities between the features of communication in humans and in social insects, though they differ in dynamics. Social insects, including termites, can be considered as a model for understanding the spread of infection via social interactions [27]. Moreover, soil invertebrates can contribute to the preservation and distribution of viruses [28].

In the current work, we studied the viromes of *H. bicolor*, *M. carbonarius* and *O. wallonensis* and found four full genomes of the novel viruses. All novel viruses were close to the viruses previously identified in the metagenomes of the termites [3,7]. Nevertheless, all novel viruses had significant differences from the known viruses. Thus, this work contributes to the description of the biodiversity of the viruses. Overall, four novel viruses were discovered in the work. Two of them (CHPLV and CHLV) were found in the pool of the *H. bicolor.* In the current work, pools of termites were studied, and we cannot make a conclusive statement about the possibility of co-infection. 

Discovered during the current work, CMSV has two segments and is related to the Kofsystermes virus, Mafsystermes virus, Wifsystermes virus and Pafsystermes virus. As of now, viruses of this group have quite different genome structure. Wifsystermes virus has the same genome structure as CMSV. Kofsystermes and Mafsystermes viruses have two segments; however, the second one is shorter (around 800 nt) and encodes only one ORF. Pafsystermes virus is reported to have only one segment with one ORF [7]. While this might indicate potential difference in the biology of these viruses, we cannot exclude the possibility of incomplete assembly of Kofsystermes, Mafsystermes and Pafsystermes viruses.

Earlier, it was suggested that Wifsystermes virus’ second ORF on the second segment requires the use of a variant genetic code, in which UGA is translated as tryptophan [7]. Here, and in our previous work [15], an alternative explanation was suggested for expression of the second ORF on the second segment in the novel solemo-like viruses. This ORF can be expressed via stop-codon codon read-through (a well-known mechanism of virus protein expression, typical for members of *Polemovirus* and *Enamovirus* (family *Solemoviridae*)) [21]. Nevertheless, direct experiments are needed to uncover the molecular biology of novel solemo-like viruses.

CHLV, detected in epiphyte-feeding *H. bicolor*, was clustered with Jimsystermes virus, previously identified in xylophagous *Occasitermes* sp., collected in Australia [7]. The next closest relative virus, Isopteran arli-related virus, was isolated from xylophagous *Coptotermes* sp., collected in Ivindo National Park (Gabon) [23]. As all these Lispiviridae viruses were detected in termites of different feeding types and on different continents, we could not determine any relationship between Lispiviridae virus presence and termite diet or habitat. Due to the association of the Lispiviridae with insects [7], we suggest that CHLV infects termites, not their feeding substrates or microbial symbionts.

CHPLV, clustered with Nuksystermes virus in the *Polycipiviridae*, forms a monophyletic group with the genus *Sopolycivirus* (Figure 3A), although with a relatively low support level. Viruses of the genus *Sopolycivirus* infect ants of the family Formicidae [24]. Sopolyciviruses are shown to have a significant impact on the infected ant queens. It was shown that queens infected with Solenopsis invicta virus 2 produced less brood, which may have been caused by the virus’ significant impact on gene expression [29]. We speculate that CHPLV may also affect its termite host. Therefore, further research is clearly needed to gain a better understanding of CHPLV.

In our study, delta-like virus was found in the *O. wallonensis*. Classical HDV needs a helper virus (HBV) to provide it with envelope protein [25]. Novel HDV-like agents were recently found in different organisms, such as rodents, snakes, fish, etc. However, when novel HDV-like viruses were discovered, no contigs with homology to HBV were found in the samples. It was also shown that HDV-like viruses can persist in the absence of a co-infection with HBV-related viruses [30]. It was speculated that novel HDV-like viruses may use other viruses as helper viruses [3,30]. In our study, no contigs with homology to the HBV were found in the *O. wallonensis* pool; however, we found several chuvirus-like contigs in the *O. wallonensis.* If these contigs indeed represent a virus, the existence of mixed infection of CODLV with putative chuvirus may be speculated. Nevertheless, proper studies are needed in order to unveil the biology of the HDV-like viruses. 

CODLV clustered with THDV in the phylogenetic trees. In contrast to HDV, additional ORFs were found in the THDV [3]. Whereas CODLV had some additional ORFs in its genome, they were not homologous to those found in the THDV. The likely explanation to this fact would be to assume that these novel ORFs are not functional in both THDV and CODLV. However, since these viruses are fairly distant, we cannot completely exclude the fact that at least some of them may be functional. More studies are needed to uncover the biology of novel HDV-like viruses.

Two contigs with the homology to viral polymerases of the genus *Deltaflexivirus* were found in the current study. We assumed that both of the deltaflexi-like contigs belonged to the single virus. However, our attempts to bridge the gap between deltaflexi-like contigs using PCR failed. There are several possible explanations for this. The overall presence of the virus in the pool might have been low, or these two contigs may have belonged to closely related but different deltaflexi-like viruses. It is also possible that these contigs represented endogenous virus-like elements or the virus itself was segmented.

We performed a phylogenetic analysis on the larger of the two deltaflexi-like contigs. Phylogenetically, it is close to two viruses, Agaricus bisporus virus 9 and Lentinula edodes deltaflexivirus 2, both found in edible basidiomycete mushrooms [31,32]. This is noteworthy, considering that *M. carbonarius* (where deltaflexi-like contigs were detected) is a species that cultivates basidiomycete fungi of the genus Termitomyces (Agaricales) as a food source [33]. If these deltaflexi-like contigs indeed form parts of a viral genome, then the virus is not likely to infect termites, but fungi cultivated by termites. However, other possible hosts, for instance, termites themselves or one of their endosymbionts, cannot be excluded. It may also be possible for a virus to be transmitted between termites and fungi. Such situations may have important ecological implications. However, additional research is needed to clarify the nature of deltaflexi-like contigs first.

## Figures and Tables

**Table 1:** Termite specimens used in the study.

Specie	Specimens in the Pool	Workers	Soldiers
*Hospitalitermes bicolor*	25	13	12
*Macrotermes carbonarius*	7	0	7
*Odontotermes wallonensis*	10	8	2

**Table 2:** Virus-related contigs that are not likely to be complete virus genomes.

Contig	Contig Length (nt)	Closest Relative	E Value	Query Cover	Identity
17_N1 + N237	4685	[AQM49933] Agaricus bisporus virus 9	0	96%	41%
17_N195 + N540	2470	[AQM49933] Agaricus bisporus virus 9	10^−125^	69%	45%
19_N269	730	[AUW34382] Blacklegged tick chuvirus 2	9 × 10^−90^	99%	58%
19_N173	925	[QBP37027] Lampyris noctiluca chuvirus-like virus 1	10^−84^	97%	48%
19_N752	427	[YP_009337904] Hubei chuvirus-like virus 1	2 × 10^−18^	97%	38%
19_N664	451	[QPL15312] Orthopteran chu-related virus OKIAV152	5 × 10^−13^	96%	30%
19_N348 + N818 + N877 + N365	1619	[QPL15312] Orthopteran chu-related virus OKIAV152	6 × 10^−82^	82%	41%

## Data Availability

Raw high-throughput sequencing data obtained during this study are available in the SRA database (BioProject accession number PRJNA817887). Obtained virus sequences and sequence of the deltaflexi-like contig 17_N1 + N237 were deposited in the GenBank database (accession numbers ON082761-ON082766).
